# Patients’ experience of undergoing maintenance hemodialysis. An interview study from Ethiopia

**DOI:** 10.1371/journal.pone.0284422

**Published:** 2023-05-30

**Authors:** Mignote Hailu Gebrie, Hussen Mekonnen Asfaw, Workagegnehu Hailu Bilchut, Helena Lindgren, Lena Wettergren

**Affiliations:** 1 School of Nursing, College of Medicine and Health Sciences, University of Gondar, Gondar, Ethiopia; 2 Department of Nursing, School of Nursing & Midwifery, College of Health Sciences, Addis Ababa University, Addis Ababa, Ethiopia; 3 Department of Internal Medicine, School of Medicine, College of Medicine and Health Sciences, University of Gondar, Gondar, Ethiopia; 4 Division of Reproductive Health, Department of Women’s and Children’s Health, Karolinska Institutet, Solna, Sweden; 5 Sophiahemmet University, Stockholm, Sweden; 6 Department of Public Health and Caring Sciences, Uppsala University, Uppsala, Sweden; Faculty of Medicine, Saint-Joseph University, LEBANON

## Abstract

**Background:**

People with kidney failure require renal replacement therapy in the form of dialysis or a kidney transplant for survival. Many facets of their life, both within and outside the dialysis unit, are impacted by the management of this disease. It is important to comprehend the experiences of people undergoing hemodialysis in order to improve the care provided to them. Therefore, this study aimed to explore the experiences of patients undergoing maintenance hemodialysis in Ethiopia.

**Methods:**

A qualitative descriptive study was conducted at two healthcare facilities in Ethiopia. Individual interviews with 15 participants (men and women aged 19–63), undergoing hemodialysis in Ethiopia, were analyzed using reflexive thematic analysis.

**Results:**

The analysis resulted in five themes: Feeling grateful, Facing a restricted life, a Supportive environment, Dreaming of a transplant, and Leading a hassled life. The subthemes include Trust in treatment, Faith in God, Challenging fluid and dietary restrictions, Being too fatigued to socialize, Being stigmatized, Family and social support, Supportive healthcare, Lacking a donor and sponsor, COVID-19 as a barrier, Financial constraints, Inaccessibility to care and transport and Access line implantation. Despite being dependent on a machine and having to deal with food and fluid restrictions as well as financial challenges, participants were hopeful and dreamed of a transplant.

**Conclusion:**

From the study’s participants, it was discovered that the experiences of people with kidney failure undergoing hemodialysis were generally, considerably negative narratives. Based on the results we recommend development of multidisciplinary teams to better meet patients’ physical, emotional, and social needs while undergoing hemodialysis. Such a team should also involve the patient’s family members when caring for patients on hemodialysis.

## Introduction

People with kidney failure require kidney replacement therapy in the form of dialysis or a kidney transplant for survival [1]. The number of people requiring kidney replacement therapy are increasing worldwide [2]. In the UK, more than one out of every thousand people receives this kind of care [3]. However, these treatment options are affected by limited access and/or high costs in most parts of the world. The problem is severe in low-income regions [4, 5], where hemodialysis is the most common modality of kidney replacement therapy [6, 7]. Approximately 3.9 million people with kidney failure underwent kidney replacement therapy worldwide in 2017 [8], but nearly a comparable number of people did not receive treatment due to a lack of access to dialysis and transplantation [9]. In Africa, where treatment access is limited, people are dying with end-stage kidney disease, with only 9–16% of cases receiving any treatment at all [5, 9]. In Ethiopia, the number of end-stage kidney disease patients are increasing because of increased risk factors due to lifestyle changes (the substitution of traditional plant-based diets with increased intake of animal products, saturated fats and processed foods, as well as decreased physical activity), which have contributed to an increased prevalence of high blood pressure and diabetes mellitus [10, 11]. Maintenance dialysis treatments were made available in Ethiopia in 2001. In urban areas of the country, dialysis services have grown significantly during the past ten years, especially private for-profit facilities [12]. There is one government run national kidney transplant center which started its services in 2015 in collaboration with the University of Michigan, USA. However, the center has not performed any transplants since March 2020 due to the pandemic [12, 13]. The cost for each hemodialysis session in the private sector is 2300 Ethiopian birr (equivalent to 51 USD) [14] and many people cannot afford this.

Although hemodialysis is life prolonging, it is physically and psychologically demanding. It requires access to the bloodstream through a surgically created fistula or a permanent intravenous line. Regular treatment session needs to be held, usually thrice weekly with each session lasting around 3–5 hours [15, 16]. This complex nature of the treatment procedure affects normal life and leads to a significant symptom burden [17, 18]. Consequently, people with chronic kidney disease are required to undergo a number of lifestyle changes, such as dietary and fluid restrictions, in order to accommodate their illness [19]. Moreover, the psychosocial issues that people undergoing hemodialysis must contend with are immense [20].

In recent years, there has been an increase in interest in using patient-reported outcome measures to evaluate issues such as quality of life and satisfaction with care. On the other hand, these attempts to quantify patient experiences do not offer the depth of understanding that qualitative methods can provide. Some qualitative studies have been carried out globally that aimed to understand the experiences of people living with chronic kidney disease in relation to the disease and its treatment [21–24]. According to these studies, participants are faced with multifaceted challenges due to the demanding nature of the disease in various parts of their life. These included living with uncertainty, relying on medical technology, having limited freedom of choice, financial restrictions and pursuing transplantation.

As far as research in Ethiopia is concerned, only a single published study has explored the lived experiences of twelve patients undergoing hemodialysis in a government hospital in Northwest Ethiopia [25]. It is important to develop a deeper understanding of the experiences of these patients in different settings so as to enable health professionals to provide adequate and appropriate support for patients on hemodialysis. Therefore, the aim of this study was to explore patients with kidney failure experiences of undergoing hemodialysis in Ethiopia.

## Methods and materials

### Study design and setting

A qualitative descriptive study was conducted at two health care facilities in Addis Ababa, Ethiopia. We used the consolidated criteria for reporting qualitative research (COREQ) checklist [26]. The filled COREQ is attached as a supplementary file (S1 Checklist).

### Study participants

Participants in this study were adult men and women (≥18 years) with kidney failure who had been on hemodialysis for three months and more at selected hospitals/dialysis centers. Purposive sampling was used, in an effort to include participants representing a diversity in terms of sex, educational status, type of hospital and duration of dialysis. Sample size was determined according to information power considering the study aim, sample specificity, theoretical background, quality of dialogue, and strategy for analysis [27].

### Procedure

The interviews were conducted in a place that was convenient for the participants when just the participant and interviewer were present. That is, at the health facility during the first hour of dialysis, at their home and in a separate area of the hospital. Of the sixteen individuals approached, fifteen consented and one patient declined to take part in the study. With the help of the head nurse of the hemodialysis unit, participants were identified from the hemodialysis unit’s patient registration book and hemodialysis procedure schedule.

### Data collection

Individual face-to-face interviews were conducted using a semi-structured interview guide (S1 and S2 Files, English and Amharic language). Before the actual interview, the guide was tested on one participant undergoing hemodialysis. After securing written informed consent, interviews were conducted in December 2021 by one of the authors (MH). Preceding the interview, demographic data (age, sex, marital status, level of education, monthly income, underlying disease, and occupation) were collected. The interviews were audio recorded using digital devices, and MH took notes, including memos about the participants’ behavior and contextual information, to supplement the data. Each interview lasted between 20 and 50 minutes.

### Data analysis

After repeated listening, all interviews were transcribed verbatim by the principal investigator and translated into English by an expert fluent in both languages for data analysis. The data were analyzed using reflexive thematic analysis. We used the most widely accepted framework for conducting reflexive thematic analysis, which involves a recursive six-phase process developed by Braun and Clarke in 2006 [28] and updated in 2019 [29]. All data processes were handled manually. The translated transcripts were repeatedly and carefully read to gain a sense of familiarization with the whole dataset. Codes were assigned to the words, phrases, sentences, and memos articulated by the respondents that were pertinent to the study. The various codes were organized in to initial themes, and all the relevant coded data extracts within the identified themes were gathered. Then, MH cross-checked the themes that was generated after the analysis of the raw data and the respective quotes in each thematic category. Appropriate names or labels were given to each theme and sub-theme. Finally, the researcher wrote description for each theme. Direct quotes from the participants were included in the write-up of the findings.

### Ethics approval and consent to participate

The study was approved by the institutional review board of the University of Gondar, St. Paul’s Hospital Millennium Medical College (No. V/P/RCS/05/2271/2020, PM23/341) and the City Government of Addis Ababa Health Bureau, (No. A/A/H/5676/227), respectively. Potential participants were given written and oral information in Amharic about the aim and procedures of the study. It was stressed that participation was voluntary and that non-participation would not affect care and treatment in any way. Informed consent was obtained from all participants and those participants who could not provide consent in writing were asked to do so through thumb print.

### Inclusivity in global research

Additional information regarding the ethical, cultural, and scientific considerations specific to inclusivity in global research is included in the Supporting Information (S2 Checklist).

## Results

Fifteen participants, nine men and six women aged between 19 and 63 were interviewed. In terms of education, the participants ranged from primary education to a college diploma and above; ten were married, and hypertension was the most common self-reported underlying disease causing chronic kidney disease. The duration of hemodialysis ranged from 6 to 72 months. Participants travel a minimum of 4 km and a maximum of 80 km to the hospital or dialysis facility. The participant’s monthly income was between 3000–15000 ETB (≈55–280 USD) (Table 1).

**Table 1:** Characteristics of participants undergoing hemodialysis.

Variables	Category	Numbers (n)
Sex	Male	9
	Female	6
Residence	In Addis Ababa	11
	Outside Addis Ababa	4
Marital status	Married	10
	Single	5
Educational status	Primary	3
	Secondary	7
	Diploma and above	5
Occupation	Employed	2
	Unemployed	13
Funding/Sponsor	Yes	5
	No	10
Underlying disease	Diabetes	5
	Hypertension	10
Duration of hemodialysis	Mean 31.2 months	
Monthly income	Mean = 7,466 birr (139 USD)	
Distance from home to hospital in kilometer	Mean = 22.1km	

In the present study, five main themes and twelve sub-themes emerged inductively from the data. The five main themes are Feeling grateful, Facing a restricted life, a Supportive environment, Dreaming of a transplant and Leading a hassled life (Table 2).

**Table 2:** Themes and sub-themes.

Themes	Sub-themes
Feeling grateful	Trust in treatment
	Faith in God
Facing a restricted life	Challenging fluid and dietary restrictions
	Being too fatigued to socialize
	Being stigmatized
A supportive environment	Family and social support
	Supportive healthcare
Dreaming of a transplant	Lacking a donor and sponsor
	COVID-19 as a barrier
Leading a hassled life	Financial constraints
	Inaccessibility to care and transport
	Access line implantation

### Feeling grateful

Feeling grateful reflect attributes related to participants’ positive attitudes towards hemodialysis and spiritual practice. The theme is comprised of two sub-themes, a trust in treatment and faith in God.

#### Trust in treatment

Participants expressed having trust in the hemodialysis treatment. They had a positive attitude toward hemodialysis and acknowledged and appreciated how the treatment helped them live longer and better lives: *“I become well and fit enough to work for at least two or three days when I have dialysis”* (Participant 7). They felt a sense of certainty that, without hemodialysis, their lives would have been miserable and, ultimately, they would have died sooner. Participants viewed dialysis as equal to their kidney, life support, a pain reliever, a means of sustaining life, a means of survival and restoring functions, which made them hopeful.

*“Dialysis meant everything for us*. *We can only do our daily activities because of the dialysis*. *It will continue to be our way of life until we get a transplant*. *We can’t survive without it*. *I now feel safe because of it*.*”*

(Participant 4)

The participants recognized that hemodialysis prolonged their lives and that, if they missed a few sessions, toxins would build up in the blood, which could cause more damage or death.

*“The benefit of having dialysis is that it would remove the excess fluid in your body*, *which you can’t naturally do*. *Otherwise*, *the fluid would make it hard for you to breathe*, *and it makes you feel uncomfortable*. *You could imagine how hard it could be to be out of breath*.*”*

(Participant 1)

#### Faith in god

The participants stated that acceptance of the reality of their condition and spirituality have a positive impact on their lives. They believed that it was God who had the power to heal or kill, not the disease: *“I myself have never thought that I would make it this far*, *for one year*. *It’s all up to God”* (Participant 6). As a result, not worrying, praying, and having faith in God were mentioned as strategies for leading a positive life. One of the participants explained that *“*…*Whether you live long or not is up to God*. *I have never thought I would survive the last two years*. *Life was tough but I had hope and believed there is nothing God wouldn’t do*.*”* (Participant 7)

### Facing a restricted life

Participants described their regret over how the kidney disease and hemodialysis had restricted their lives. Hemodialysis was seen as a hindrance to the desired way of life. Participants described that they underwent hemodialysis due to lack of options or as an alternative until they met the requirements for kidney transplant. Challenging fluid and dietary restrictions, being too fatigued to socialize and being stigmatized are the sub-themes that were identified.

*“Had I had the transplant*, *there would have been nothing I would complain about and would have been relieved from pain and physical limitations*… *I pity myself when I see children run here and there*.*”*

(Participant 8)

Most participants expressed that an unwanted dependency on the hemodialysis machine, feeling inferior and losing freedom were the unfavorable side of the disease and its treatment.

*“It has been 3 years since I am on dialysis*. *I am also waiting to have a transplant*. *Having dialysis is very tough*. *It feels like you are inferior to normal people*. *You wouldn’t be able to study*, *relax and eat food of your choice*.*”*

(Participant 11)

#### Challenging fluid and dietary restrictions

Challenging fluid and dietary restrictions were mentioned by almost all the study participants. They described that they had made a significant transition in their lifestyle related to fluid and dietary intake. Despite the unpleasant experience, most participants strictly adhered to the fluid and dietary recommendations. They believed that self-control and acceptance of their condition were their reasons for surviving such a restricted life.

*“I also drink little water*, *not much*. *The healthcare professionals have also advised me to eat anything except salt*. *Of all types of vegetables and fruits*, *I am also allowed to eat only apple*. *Because it is an obligation*, *I only stick to the food items that I am allowed to eat and save myself from harm*.*”*

(Participant 2)

In contrast, some participants believed that tolerating the restrictions was a challenge and failed to comply with them.

*“It is hard to give up a habit that you are told to refrain from*. *Accordingly*, *I sometimes feel thirsty and drink water*. *That is one of the worst parts of the disease*. *I often fail to resist the urge to drink water and end up drinking*. *When I do*, *I will have a very bad night*.*”*

(Participant 13)

#### Being too fatigued to socialize

Participants experienced fatigue due to unpleasant sensations such as feeling sick, being weak and not wanting to get out of bed during and after having dialysis. As a result, they usually preferred to stay at home and remain isolated from participating in social life.

*“I am unable to spend time with my friends*, *because I am usually fatigued*, *so I stay home all the time*. *I don’t go out*. *I am not pursuing my education as well*.*”*

(Participant 11)

The participants acknowledged that having kidney disease and undergoing treatment with hemodialysis had changed their social and occupational lives. They believed that their condition had prohibited them from doing what they wanted to do. In addition, their sexual desires and marital relationships had been affected.

*“I often feel fatigued and don’t make love to my wife*. *Also*, *there are many other challenges that have horizontal and vertical effects*.*”*

(Participant 6)

#### Being stigmatized

The participants complained that they were suffering from stigma and discrimination on the part of their family and society. They believed that people who used to spend time with them while they were healthy were no longer in touch once they learned of their condition. One of the participants said that *“People used to come and visit me before*. *After I got sick*, *no one seemed to care*. *They avoid you*, *even if you try to socialize*.*”* (Participant 9)

Participants also expressed that people had abandoned them due to their illness.

*“There was no one to visit me at the hospital back then (diagnosed with kidney failure)*. *People including my family abandoned me when they knew about me having a kidney failure*.*”*

(Participant 1)

### A supportive environment

To attain a desirable outcome, the support of family and friends, as well as a smoothly running healthcare system and kind healthcare workers, is required. In this regard, family and social support and supportive healthcare were identified and are described below.

#### Family and social support

The participants stated that support from family, friends and others, including financial, physical and companion support, plays an irreplaceable role in the sustainability of their treatment and their emotional strength.

*“I don’t remember a time when I have addressed any challenge on my own*. *My husband and family have done and are doing whatever they can*. *My husband is always there with me*. *Also*, *I haven’t observed any feeling of fatigue amongst them*.*”*

(Participant 10)

#### Supportive healthcare

Participants described that they had a good relationship with the dialysis center staff and that they were receiving kind care, which comforted them when they came for their routine treatment.

*“We have a good and healthy relationship with the healthcare professionals*. *They are very supportive and ready to supply us with the information we need*. *They treat us better than we treat ourselves*. *They know what we need and what we have to do*. *They encourage us to feel free and ask whatever we want*.*”*

(Participant 12)

Moreover, participants perceived that the healthcare services they were receiving from government dialysis centers were far better than private services in terms of quality and affordability. Participants appreciated the material-handling techniques and the compassionate care offered by care providers in public institutions.

*“*…*therefore*, *I say that there is a big difference in terms of quality of service between governmental and private health institutions*. *I have had dialysis in a private health institution for 6 months*, *and I have come to observe the difference*. *The quality of the service is far better in governmental institutions*. *Thus*, *it would be better to extend and develop the service in the governmental institutions*.*”*

(Participant 3)

### Dreaming of a transplant

Participants described a kidney transplant as a means to improve their quality of life given the challenges they confront in hemodialysis. Dreaming of a transplant included two subthemes: lacking a donor and sponsor and COVID-19 as a barrier. The participants in this study experienced difficulty in finding a donor and sponsor for transplantation. They also described COVID-19 as the reason for the closure of the transplantation service.

#### Lacking a donor and sponsor

The participants noted two reasons for not having a timely kidney transplant (the lack of a donor and sponsor). Almost all participants described that they wanted to have a kidney transplant. However, the issue of finding donor and sponsor was the key to making their dream come true. Therefore, most of the participants were simply dreaming of having the transplant until they could meet these requirements.

*“Regarding a transplant*, *1) it requires money*, *and 2) it requires a donor*. *These two are very crucial for it*. *Maybe*, *you have a donor*, *and you don’t have money to facilitate the transplant because you are aware it’s done in other country that way and the option is to be on dialysis until*… *I may have a donor from the family because I have brothers*, *but maybe I don’t have money to do this*. *Currently*, *I have a sponsor paying for my dialysis*, *but they can’t pay money for transplantation abroad*.*”*

(Participant 14)

#### COVID-19 as a barrier

The emergence of the COVID-19 pandemic was described by many participants as a barrier to a timely kidney transplant. The participants described that they were eagerly waiting for the transplantation service, which had been locked down due to the pandemic, to resume.

*“I was almost on the verge of having a transplant*. *First of all*, *the reason as to why I came here was that I was entitled to have a transplant*. *Unfortunately*, *it was the time of COVID-19*, *and they had to suspend the transplant and hadn’t resumed doing transplants*. *I am amongst the first patients on the list waiting to have a transplant*.*”*

(Participant 13)

### Leading a hassled life

Participants addressed that life in some ways was difficult as a result of the disease and its treatment. Several hassles were addressed in the interviews and they are summarized in three subthemes: financial constraints, inaccessibility and access line infection and inconvenience.

#### Financial constraints

Limited financial resources were mentioned as the main hassle in receiving dialysis services by the participants involved in this research. They talked about their financial struggles in covering their medical expenses. A lack of sustainable income or a sponsor, unemployment due to their condition and unaffordable costs were reported as barriers to the service. In addition, financial constraints and the unaffordability of the service were mentioned as reasons for noncompliance with the recommended frequency of dialysis and supporting drugs. Financial constraints were mentioned as a distressing experience by most participants.

*“The doctor has recommended me to have dialysis three times a week*, *but I could only afford one or two treatments per week*. *That*, *of course*, *has an effect*. *Still*, *the treatment is very expensive*, *and I can’t afford it*. *Thank God I am in better condition compared to those patients who couldn’t even afford a single treatment a week*. *A single treatment costs 3*,*000 birr (55 USD)*, *and many patients become stressed*. *For one thing*, *it is shameful to always ask people for help*. *For the other*, *you can’t work and earn money*.*”*

(Participant 7)

The participants also mentioned that their condition and its costly treatment had caused them to lose the wealth they had earned over their lifetimes. Many had sold out their houses, cars and plots of land to cover the costs of their treatment; some begged for money from family members and others.

*“The financial problem is immense*. *I myself had to sell two pieces of plots of land in the past year*.*”*

(Participant 15)

#### Inaccessibility to care and transport

Service inaccessibility is one of the barriers to obtaining hemodialysis service in a timely fashion. The limited number of facilities, a lack of transportation and distance were mentioned as some of the barriers in terms of accessing the service.

Many participants travel long distances two or three times each week to receive hemodialysis. They suffer in finding transport to reach to a hemodialysis center and considering their dialysis schedule.

*“The challenges*, *including transportation*, *are many*. *For instance*, *I was on the morning shift today*, *yet I was unable to find transportation and called my friend to change shifts with me*. *I would still have a problem in finding a taxi to get home*, *so it will be late for me to go home*.*”*

(Participant 4)

The participants stated that they moved from where they lived with their family to Addis Ababa, where they now live lonely lives. This situation caused them lose their job and created an additional financial burden for their families.

*“It has been 2 years since I last saw my family and stopped doing my job*. *What is difficult is that there is no such treatment where some patients come from*, *and when they come to other places to have dialysis*, *they lose their jobs and miss the life they had*.*”*

(Participant 13)

The participants stated that concerns such as machine breakdown, a lack of filtered water and other supplies, which contribute to interruption of the hemodialysis services, are common problems they face. As a result, they are forced to spend long hours or even a number of days in the center.

*“Sometimes*, *we would have to spend the night here*, *when a machine breaks down*. *For example*, *we are on the third shift today*, *and we have to wait because only two of the machines are functional*. *When that is the case*, *we often come to terms with one another and try to have the dialysis*.*”*

(Participant 4)

#### Access line implantation

The participants stated that access line implantation is another hassle faced during hemodialysis treatment. Most participants preferred an arteriovenous (AV) fistula in an arms to a catheter on their necks. They believed that the catheter was not convenient in terms of moving their neck and also more prone to infection than the fistula.

*“The difficult part about dialysis is that you need to get an access line implanted in your body*. *It may be in your neck or arm*. *The access line you get in your arm is relatively safe*. *I was*, *anyway*, *afraid to have one of the two alternatives until it became a must to have it*. *Similarly*, *after I got the access line in me*, *it became infectious and caused me a lot of suffering*. *Accordingly*, *it used to take me long time of treatment*.*”*

(Participant 1)

## Discussion

The present study investigated patients’ experiences of undergoing maintenance hemodialysis in Ethiopia. The thematic analysis resulted in five main themes: Feeling grateful, Facing a restricted life, Supportive environment, Dreaming of a transplant and Leading a hassled life. Comparing our study to previous reports from other countries reveal that the experiences of Ethiopian patients and patients on hemodialysis in other nations have a great deal in common [21, 23, 24].

Trust in treatment was evident in this study. Similarly, according to a study conducted in the UK, participants had a positive attitude toward hemodialysis because they believed that hemodialysis was a lifeline and that it was psychologically beneficial to ‘live in the moment’[24].

Our findings revealed that the participants had a strong faith in God. This is in line with a study conducted in Jordan and India in which faith in God and religion were the most commonly practiced coping strategies among patients on hemodialysis [30, 31]. Similarly, a systematic review reported that spirituality enables hemodialysis patients to give value to their lives and feel strong while dealing with this challenging disease [32]. A similar study performed in Ghana is also consistent with our findings in that participants exhibited spiritual fluctuation (belief in God and slips of faith) [33]. They described belief in God as a means of accepting and coping with the disease. Thus, it may be helpful to consider religious persons in the care provision of patients on maintenance hemodialysis.

Even though the participants believed that the machine was equal to their kidney, they experienced changes in their everyday lives, including unwanted dependence on the hemodialysis machine, feeling inferior, losing freedom and difficulty in maintaining their preferred way of life. Such feelings are consistent with studies conducted in the UK, Thailand and Jordan [15, 18, 24].

Challenging fluid and dietary restrictions related to patients on hemodialysis were frequently addressed. Studies conducted in India, Georgia, Jordan and Ethiopia [23, 25, 31, 34] have also demonstrated that individuals in hemodialysis treatment report having to limit their intake of food and fluids, indicating that they feel constrained regarding their ability to eat the foods they want. Therefore, the dialysis team should design a sustainable teaching program concerning how self-control regarding fluid and food restrictions is significant for the patient’s life to help them adhere with such recommendations. In line with this observation, a study conducted in Iran revealed that educational intervention significantly increased the fluid and dietary adherence of hemodialysis patients [35].

In this study, participants experienced fatigue, which hindered their attempts at routine social activities, in line with results of previous studies [22, 33, 36, 37]. Similarly, participants in this study revealed that fatigue altered their jobs, sexual desires and marital relationships, which is in line with the finding of studies in Jordan, Spain and Ethiopia [25, 38, 39].

According to this study, participants felt stigmatized by their family and the community, which is congruent with a similar study performed in Ghana [33], in which people undergoing hemodialysis received unwelcomed treatment from their family and friends, including refusal to eat with the participants.

In the current study, many participants expressed the importance of family and social support to adhere with their treatment Several studies reflect similar results, explaining how family and social support helps in dealing with this disease [25, 31, 37, 40, 41]. Companionship and emotional support while meeting the patients’ daily needs are critical factors to consider when designing a care plan for these patients. Thus, nurses are encouraged to include family members throughout the entire treatment planning, assessment, implementation and evaluation phases in order to best address patient needs and increase their well-being. Furthermore, in a case such as broken family trust and difficulties adjusting to disease condition, nurses can be of great assistance in mitigating the effects.

Patients’ overall perception of healthcare is impacted by their relationship with their healthcare team. Another important finding of this study is supportive healthcare, which is in line with a study performed in Iran [40], in which participants expressed that they found comfort and assurance in the positive relationships they have with the nurses and doctors. An Indian study also demonstrated supportive healthcare in that the participants said they had received support from doctors and were satisfied with nursing care [31]. In addition, the participants in the current study expressed their trust in government-owned institutions and praised the care they had received from them. Though there is no supporting literature, the finding is seen may be due to the presence of participants who have been served in both public and private institutions. Therefore, it would be commendable to expand on this aspect of the problem to determine whether one has something to learn from the other.

The present study revealed that participants dreaming of a transplant despite a lack of donors and sponsors, as well as the emerged global pandemic COVID-19, were still described as barriers. This finding is congruent with those of a study conducted in the UK [24], in which some individuals looked forward to kidney transplants for the purpose of increasing the normalcy of their lives. However, they have been challenged in finding suitable and willing donors. Community-based teaching initiatives may be more effective at increasing donation rates.

Participants disclosed their suffering while finding transport to and from the hemodialysis center reflected in the sub-theme ‘inaccessibility to care and transport’. This finding is in agreement with a study in the US in which the issue of transportation was a major challenge for dialysis service adherence [42]. According to the present study, it is difficult for these participants because the treatment requires them to travel to and from the dialysis center three times per week. Moreover, some of the participants in the present study were not residents of Addis Ababa; they traveled from cities up to approximately 90 km from Addis Ababa, to attend hemodialysis. The government must take into account offering a public transportation system or other assistance to people who live in cities without access to dialysis centers. The results of the present study indicate that, because the available hemodialysis centers in the country are concentrated in the capital, Addis Ababa, participants opted to displace themselves from their permanent residence to access treatment. Building more hemodialysis centers, especially in the main cities of Ethiopia, could help ease the difficulties that people on dialysis who live outside of Addis Ababa face in accessing these services and lessen the strain that the dialysis centers in the capital city are under.

Hemodialysis is a costly treatment. In the present study, participants disclosed their worry concerning financial constraints and an absence of income in relation to job discontinuation as a result of the disease and its treatment. Participants frequently chose to reduce the number of hemodialysis sessions per week, skipped taking their prescribed medications and sold their properties due to financial limitations. This finding is consistent with studies conducted in Nigeria, Ethiopia, Ghana, Jordan and the US [22, 25, 33, 34, 36]. There is a need for the Federal Ministry of Health to increase the number of government-funded dialysis centers and monitor the cost and quality of renal services provided in the country to reduce the arbitrary prices for dialysis seen in private dialysis facilities.

The participants in this study explained that using catheter as an access line was more inconvenient and prone to infection than AV fistula. This finding is supported by a previous study that revealed the use of a catheter is associated with a high risk of complications including infection [43].

### Strengths and limitations of the study

In terms of a strength of this study, the diverse perspectives of those receiving care at various locations were included by recruiting participants from both private and public dialysis facilities. We used Guba’s criteria to ensure the trustworthiness and rigor of the data [44]. Credibility was achieved through investigator triangulation and participant validation. All of the team members were involved from the conception of the project, data analysis (MHG, LW, HL) and interpretation to write-up. To promote confirmability, the thorough methodological description we used will enable the reader to determine the acceptability of the data and the constructs resulting from them. Dependability was improved via a detailed description of the methodology, as well as how the findings were reached and whether they were ethically sound. To ensure data transferability, we recruited participants who were experienced about the phenomenon being studied, with the maximum variety, and obtained a thick and detailed interpretative description of the phenomenon. The limitation, however, is that the translation of the original Amharic version data to English for analysis and write-up might have introduced deviation in some contextual expressions of the participants.

## Conclusion

The experiences of patients receiving maintenance hemodialysis in capital of Ethiopia is not unique, it has many things in common with previous research conducted in other countries. Despite being dependent on a machine and having food restrictions as well as financial challenges, participants were hopeful and dreamed of a transplant. The findings may increase healthcare providers’ understanding of Ethiopian patients undergoing hemodialysis. Based on the results we recommend development of multidisciplinary teams to meet the individual’s physical, emotional, and social needs while undergoing hemodialysis. Such a team should also involve the patient’s family members when caring for patients on hemodialysis.

## Supporting information

S1 File English version interview guide. (PDF)

S2 File Amharic version interview guide. (PDF)

S1 Fig Coding tree. (PDF)

S1 Checklist Filled Consolidated Criteria for Reporting Qualitative Studies (COREQ) checklist. (PDF)

S2 Checklist Inclusivity in global research. (DOCX)
