# Supplementary material for: Patients’ experience of undergoing maintenance hemodialysis. An interview study from Ethiopia
Source: PLoS One. 2023 May 30;18(5):e0284422. doi: 10.1371/journal.pone.0284422 (PMC10228775; doi:10.1371/journal.pone.0284422)
Supplement: S1 File — (PDF) [file pone.0284422.s001.pdf]

Guiding questions for the in-depth interview

1. So you are now getting treatment for a kidney disease, tell me more about that?
2. Tell me what it is like for you to be on hemodialysis?
3. Tell me what it is like for you to take your medications as prescribed?
4. I have understood that you are recommended to have food and fluid restrictions, tell me more about that?
5. How do you communicate with the health care providers?
6. Describe how it was to choose hemodialysis from the different treatment options for your kidney disease??
7. Based on your experience, what would you tell someone new to dialysis about the challenges of their treatment?
8. Is there anything else you would like to say about your experience as a person on hemodialysis?
