# Supplementary material for: Patients’ experience of undergoing maintenance hemodialysis. An interview study from Ethiopia
Source: PLoS One. 2023 May 30;18(5):e0284422. doi: 10.1371/journal.pone.0284422 (PMC10228775; doi:10.1371/journal.pone.0284422)
Supplement: S2 File — (PDF) [file pone.0284422.s002.pdf]

ለጥልቅ ቃለ መጠይቁ መሪ ጥያቄዎች

1. እንግዲህ አሁን ለኩላሊት ህመምዎ ህክምና እያገኙ ነው፣ እስኪ ስለሱ በደንብ ይንገሩኝ?
2. ሄሞዳያሊስስ ላይ መሆን ለርሰዎ ምን እንደሚመስል እስኪ ይንገሩኝ?
3. መድሃኒትዎን በታዘዘው መሰረት መውሰድ ለእርስዎ ምን እንደሚመስል ይንገሩኝ?
4. የምግብ እና ፈሳሽ ነገሮች አወሳሰድ ገደብ እንዲኖርዎት እንደሚመከሩ እረዳለሁ፣ እስኪ ስለሱ የበለጠ ይንገሩኝ?
5. ከጤና ባለሙያዎች ጋር ያለዎት መግባባት እንዴት ነዉ?
6. ለኩላሊት በሽታዎ ከተለያዩ የሕክምና አማራጮች ሄሞዳያሊስስን እንዴት እንደመረጡ ይግለጹልኝ?
7. ካለዎት ልምድ በመነሳት ለዳያሊስስ አዲስ ለሆነ ሰው ስለ ህክምናው ተግዳሮቶች ምን ይነግሩታል?
8. እንደ አንድ ሄሞዳያሊስስ ላይ እንዳለ ሰው ሄሞዳያሊስስን በተመለከተ ስላለዎት ልምድ ለመናገር የሚፈልጉት ሌላ ነገር አለ?
