# Supplementary figures and images for: Patients’ experience of undergoing maintenance hemodialysis. An interview study from Ethiopia
Source: PLoS One. 2023 May 30;18(5):e0284422. doi: 10.1371/journal.pone.0284422 (PMC10228775; doi:10.1371/journal.pone.0284422)

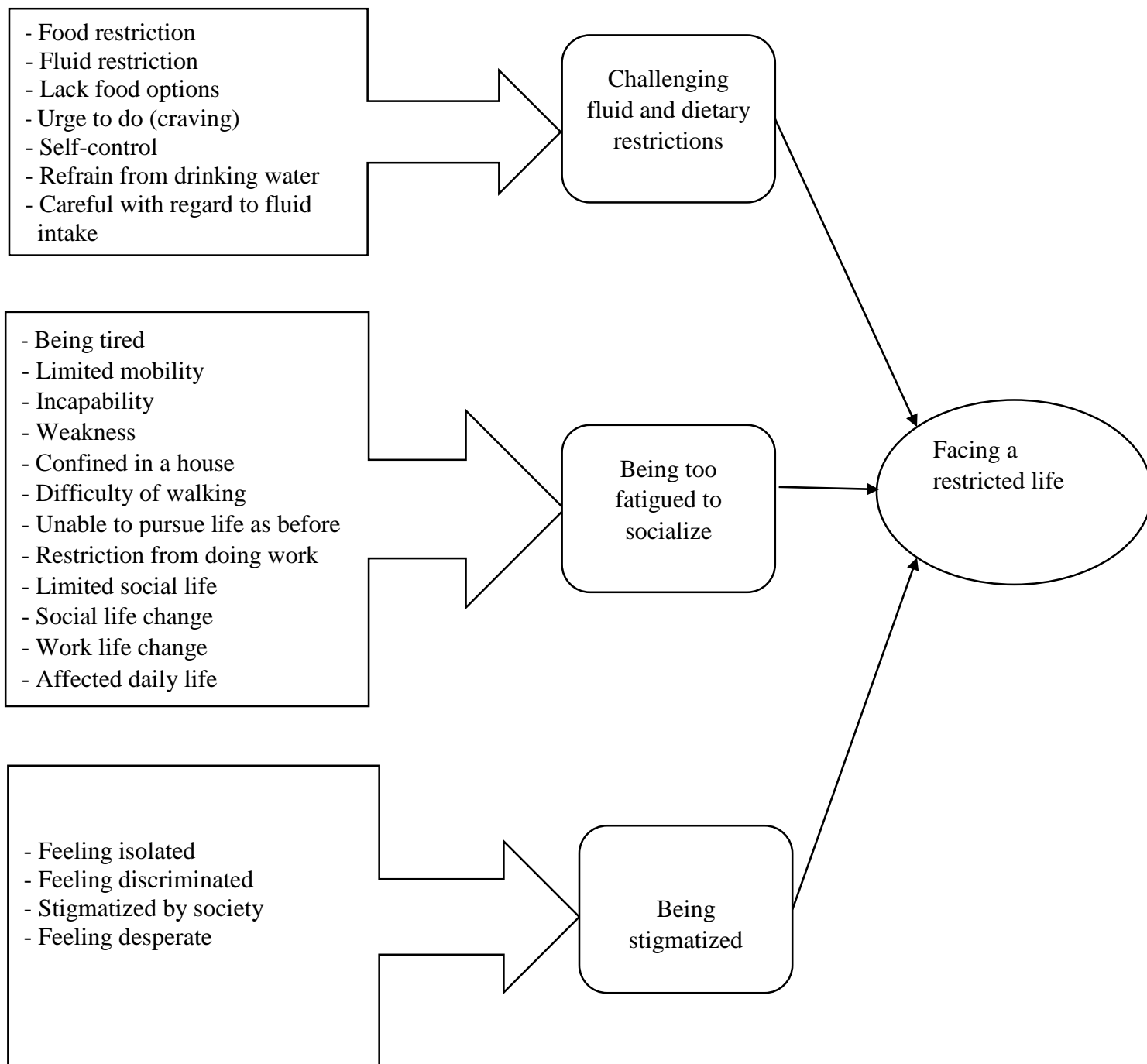

Figure 1: Sample coding tree

Supplement: S1 Fig — (PDF) [file pone.0284422.s003.pdf]
