# Supplementary material for: Patients’ experience of undergoing maintenance hemodialysis. An interview study from Ethiopia
Source: PLoS One. 2023 May 30;18(5):e0284422. doi: 10.1371/journal.pone.0284422 (PMC10228775; doi:10.1371/journal.pone.0284422)
Supplement: S1 Checklist — (PDF) [file pone.0284422.s004.pdf]

Filled Consolidated Criteria for Reporting Qualitative Studies (COREQ) checklist

**Topic**	**Item No.**	**Guide questions/Descriptions**	**Reported on page No.**
**Domain 1: research team and reflexivity**			
Personal Characteristics			
Interviewer/ facilitator	1	Which authors conducted the interview or the focus group?	MH carried out the interviews (page 5)
Credentials	2	What were the researcher's credential's?	MH has MSN, LW, HM, and HL each have a Ph.D., WH is a nephrologist (page 23)
Occupation	3	What was their occupation at the time of the study?	MH is a student, LW, HL, HM and WH are a professor in University (page 23)
Gender	4	Was the researcher male or female?	MH, LW and HL are female. WH and HM are male (page 23)
Experience and training	5	What experience or training did the researchers have?	MH joined training for research methodology and qualitative study during her period of Ph.D. study. LW, HL and HM had previous experience of this methodology
Relationship with participants			
Relationship established	6	Was a relationship established prior to the study commencement?	No prior relationship was established between the researchers and participants
Participant knowledge of the interviewer	7	What did the participants know about the researcher? E.g. personal goals, reasons for doing research	Participants were briefed on the purpose of the study and understood that it was a research project. Ethical approval had been granted, participants reviewed the participant information sheet prior to giving written informed consent to be involved (page 6-7)
Interviewer characteristics	8	What characteristics were reported about the interviewer/facilitator? E.g. bias, assumptions, reasons and interests in the research topic	The three researchers had an interest in the research. (page 4)
**Domain 2: study design**			
Theoretical framework			

Methodological orientation and theory	9	What methodological orientation was stated to underpin the study? E.g. grounded theory, discourse analysis, ethnography, phenomenology, content analysis	Reflexive thematic analysis (page 6)
Participant selection			
Sampling	10	How were participants selected? E.eg. purposive, convenience, consecutive, snowball	A purposive sample of 15 participants was recruited (page 5)
Method of approach	11	How were participants approached? E.g. face to face, telephone, mail, email	A face-to-face interview was carried out (page 5)
Sample size	12	How many participants were in the study?	15 participants took part in the study (page 5)
Non-participation	13	How many people refused to participate or dropped out? Reasons?	Of the 16 people being contacted, 15 participants responded and were successfully interviewed.<br><br>Reasons: Our ethical clearance did not approve collecting such data
Setting			
Setting of data collection	14	Where was the data collected? E.g. home, clinic, workplace	In the health facility, the patient's home (page 5)
Presence of non-participants	15	Was anyone else present besides the participants and researchers?	No, only the interviewer and the patient (page 5)
Description of sample	16	What are the important characteristics of the sample? E.g. demographic data, date	Age range from 19 to 63. Nine males and six females. Participants with primary education to a college diploma and above were included (page 6)
Data collection			
Interview guide	17	Were questions, prompts, guides provided by the authors? Was it pilot tested?	Interview guide was used. Follow-up questions were allowed. These were extensively reviewed by the research team (page 5)
Repeat interviews	18	Were repeat interviews carried out? If yes, how many?	No
Audio/visual recording	19	Did the research use audio or visual recording to collect the data?	Yes, interviews were audio-recorded (page 5-6)
Field notes	20	Were field notes made during and/or after the interview or focus group?	Yes (page 5)
Duration	21	What was the duration of the interviews or focus groups?	Interviews ranged from 20 to 50 minutes (page 6)

Data saturation	22	Was data saturation discussed?	No, we used information power to determine the sample
Transcripts returned	23	Were transcripts returned to participants for comment and/or correction?	No
**Domain 3: analysis and finding**			
Data analysis			
Number of data coders	24	How many data coders coded the data?	One (page 6)
Description of the coding tree	25	Did authors provide a descriptions of the coding tree?	Yes, a coding frame was provided in S3_Fig.
Derivation of themes	26	Were themes identified in advance or derived from the data?	Themes were derived from the data (page 8)
Software	27	What software, if applicable, was used to manage the data?	No software used
Participant checking	28	Did participant provide feedback on the finding?	No
Reporting			
Questions presented	29	Were participant quotations presented to illustrate the themes/findings? Was each quotations identified? E.g. participant number	Yes, quotations with participant numbers have been presented throughout the result section (pages 9-17)
Data and finding consistent	30	Was there consistency between the data presented and the findings?	Yes, We tried to report the study findings in a clear, consistent manner in order to accurately reflect the data that have been collected
Clarity of major themes	31	Were major themes clearly presented in the findings?	Yes, major themes are clearly presented in the result section
Clarity of minor themes	32	Is there a descriptions of diverse cases or discussion of minor themes?	Yes, minor themes are discussed
